# Clinical outcomes with distance‐dominant multifocal and monofocal intraocular lenses in post‐LASIK cataract surgery planned using an intraoperative aberrometer

**DOI:** 10.1111/ceo.13153

**Published:** 2018-02-23

**Authors:** Bret Fisher, Rick Potvin

**Affiliations:** ^1^ Eye Center of North Florida Panama City Florida USA; ^2^ Science in Vision Akron New York USA

**Keywords:** cataract surgery, intraoperative aberrometry, LASIK, multifocal IOL, refractive surgery

## Abstract

**Importance:**

Studies evaluating the clinical benefits of intraoperative aberrometry (IA) in cataract surgery are limited.

**Background:**

The study was designed to determine whether IA improved clinical outcomes of post‐laser *in situ* keratomileusis (LASIK) cataract surgery with different intraocular lenses (IOLs) implanted.

**Design:**

A retrospective chart review of clinical outcomes from one surgeon at one surgical centre was conducted. It included post‐LASIK cataract surgeries where IA was used for the confirmation of IOL power, with either a distant‐dominant multifocal IOL or a monofocal IOL implanted.

**Participants:**

Records for 44 eyes of 31 patients were analysed.

**Methods:**

Differences in visual acuity (VA) and refractions by lens type were compared, and the effects of IA were evaluated.

**Main Outcome Measures:**

Uncorrected distance VA and the percentage of eyes with a spherical equivalent refraction within 0.5D of the intended correction were the primary outcome measures.

**Results:**

There was no statistically significant difference in the percentage of eyes with uncorrected distance VA of 20/25 or better between IOL groups (*P* = 0.41). More eyes in the multifocal group had a refraction within 0.50D of intended (*P* = 0.03). In 39% of cases, the preoperative and IA power calculations suggested the same IOL power. When not equal, the IA results were not significantly more likely to be ‘best’ (*P* = 0.08).

**Conclusions and Relevance:**

Results suggest that a history of previous LASIK is not a contraindication to use of distant‐dominant multifocal IOLs. IA did not appear to improve clinical outcomes in post‐LASIK eyes, although a positive trend was evident.

## Introduction

Laser *in situ* keratomileusis (LASIK) patients are considered more likely to be sensitive to the reduced vision resulting from crystalline lens opacification. Studies have indicated that post‐LASIK patients undergo cataract surgery earlier than those with no history of previous corneal refractive surgery.^1^, ^2^ In addition to presenting earlier, post‐LASIK patients may also have higher expectations for their refractive outcome, hoping for the same high level of spectacle independence they enjoyed after their corneal refractive surgery.

Intraocular lens (IOL) power calculations after any refractive surgery are challenging, primarily because of the change in the ratio between the anterior and posterior corneal curvature; this alters total corneal power. The removal of corneal tissue may also affect the prediction of effective lens position.^3^ Standard IOL power calculations do not account for this, so specific methods are required to manage these eyes. This may include using specific devices to help measure the cornea and/or specific formulas that better adjust for the post‐LASIK state of the eye.^3^ There have been individual studies^4^, ^5^, ^6^ to evaluate the various formulas available, as well as a meta‐analysis of the published literature to determine the accuracy of the various formulas.^7^ One study suggests that a ray‐tracing formula could provide results in post‐LASIK eyes that are as good or better than the standard for virgin corneas.^8^ To date, there is no single formula that has been shown to be demonstrably better in all cases. The American Society of Cataract and Refractive Surgery (ASCRS) maintains a website of current IOL power calculation formulas that have demonstrated effectiveness in post‐LASIK eyes (http://www.ascrs.org).

Another approach to calculating IOL power in these eyes is to use intraoperative aberrometry (IA); this allows for direct measurement of the power of the aphakic eye. One commonly used intraoperative aberrometer is the Optiwave Refractive Analysis System (ORA System, Alcon Laboratories, Inc., Fort Worth, TX, USA), a device which is based on Talbot–Moiré interferometry. The use of the ORA System in post‐LASIK corneas has been shown to provide similarly good, and sometimes better, outcomes than those based on preoperative (Preop) measurements and calculations.^9^, ^10^

LASIK patients generally have high expectations for reduced spectacle independence after cataract surgery. As such, they are often interested in multifocal IOL implantation, although some surgeons are hesitant to use them because of the greater difficulty in achieving an emmetropic result in post‐LASIK eyes. Studies have shown that providing good distance, intermediate and near vision is possible with multifocal IOLs while still maintaining a good safety profile.^11^, ^12^, ^13^, ^14^ We are unaware of any study addressing the use of a distant‐dominant multifocal IOL in patients with a history of LASIK.

The AcrySof IQ ReSTOR +2.5D (Alcon Laboratories, Inc.) IOL is a relatively new diffractive multifocal IOL, featuring a low add power and a distant‐dominant light distribution scheme. It is a hydrophobic acrylic lens designed to provide two distinct foci, one at distance and the other at about 50 cm.^15^ When compared to monofocal lenses, the ReSTOR +2.5D lens has been shown to provide similar distance vision and improved near vision, with little difference in visual quality.^16^

The purpose of the current study was to evaluate the clinical outcomes of post‐LASIK cataract surgery performed using an intraoperative aberrometer when a monofocal or distant‐dominant multifocal IOL was implanted.

## Methods

This study involved a retrospective chart review of cases where subjects had a prior history of LASIK, operative planning using IA and were either implanted bilaterally with the AcrySof ReSTOR +2.5D multifocal IOL or with the AcrySof monofocal IOL (SN60WF lens, Alcon Laboratories, Inc.). The patients were self‐selecting for the lens implanted with the advice of the surgeon, so some bias may be expected as each lens meets the needs of different cataract patients.

Inclusion criteria included those who had uncomplicated cataract surgery or refractive lens exchange with no pathology that compromised visual acuity (VA; outside of residual refractive error); a postoperative binocular best‐corrected VA of 20/40 (0.3 logMAR) or better was required. No other corneal surgery, aside from LASIK, was permitted. In addition, refractive and VA data in the range of interest (3 months, 70–140 days) had to be available.

The proposed sample size was based on presuming that that the mean UCVA would be 0.0 logMAR for all eyes, with a standard deviation of 0.05 logMAR (1/2 line). Detecting a 1/2 logMAR line (0.05 logMAR) of VA difference between two groups would be clinically significant. If these values are normally distributed and with a power of 0.8, an independent *t*‐test would require a sample size of 17. Presuming that the use of both eyes would require a 25% increase in this sample size, the enrolment target was 21 eyes in each group.

Preop data extracted from the files included general demographic information, LASIK information (the time between LASIK and cataract surgery, along with the details of the LASIK surgery where available), pre‐cataract biometry and the calculated IOL power and residual spherical refraction expected. Operative data included details regarding any femtosecond laser use, the results of IA, IOL type implanted, the actual IOL power and any adverse events. Postoperative data included the time of follow‐up, the manifest refraction and the best‐corrected and uncorrected distance VA. For the distant‐dominant multifocal, distance‐corrected intermediate and near VAs were also measured. All Preop IOL calculations were made with the Holladay post‐LASIK formula. Residual refractive errors and VAs were compared between lens types.

The lens power(s) suggested by Preop calculations and IA were known, as was the lens power implanted. Using the latter, and the postoperative refractive error, the expected residual refractive error from the IOL implanted can be calculated for each calculation method. This is the residual refractive error predicted for the lens power from each calculation method, adjusted by the difference between the calculated lens power and the actual IOL power implanted, determined using standard back‐calculation techniques. The difference between these values and the postoperative residual refractive error in the eye was the measure of interest, and was termed the prediction error.

Standard comparisons of the prediction error by method were made. In addition, a new analytical method developed by Potvin, similar to the technique described by Davison and Potvin in an analysis of normal eyes,^17^ was used to determine the likely benefit of IA in the current study. The new method was developed to address some limitations in previous analyses. First, earlier techniques were often biased by including a given eye in a ‘Preop calculation group’, or an ‘IA group’, based on the power of the lens implanted; this had the potential to bias results depending on the choices of the surgeon. The new technique includes all eyes in both groups. Second, prior methods usually considered only the average (aggregate) differences between the predicted residual refractive error and the actual residual refractive error. The new technique considers the potential impact of IA on an eye‐by‐eye basis. The differences between the IOL power calculated preoperatively and with IA were determined. The adjusted residual refractive errors were calculated as described in the previous paragraph. The method of calculation with the lowest adjusted residual refractive error was considered ‘best’; if values for both methods were within 0.1D of each other, they were considered equal. The ratio of best by calculation method was then compared to a random outcome (i.e. 50/50) using a chi‐square test.

The accuracy of the Preop formula is, of course, an important factor in this type of analysis. To investigate the relative effects of the Preop formula, IOL power calculation results from the Barrett TrueK formula, a recognized standard for post‐LASIK calculations, were also made. These results were analysed in the same fashion as the Holladay post‐LASIK results.

Microsoft Excel and Access were used for data collection, collation and basic analyses (both Microsoft Corp., Redmond, WA, USA). Statistical analyses were performed using the Dell Statistica data analysis software system, version 13 (Dell, Inc., Round Rock, TX, USA). Statistical testing of parametric variables was performed using analysis of variance (ANOVA), while categorical variables were tested using appropriate non‐parametric tests. Statistical significance was set at *α* = 0.05.

## Results

Records for 44 eyes of 31 patients were identified that met the inclusion criteria. Table 1 summarizes the Preop data for these eyes by lens type implanted. As can be seen, the average age of patients implanted with the multifocal IOL was statistically significantly lower than for patients implanted with the monofocal IOL. Except for age, there was no statistically significant difference in the characteristics of the two IOL groups. Detailed information regarding the previous LASIK was not available, but the type of LASIK (myopic/hyperopic) was identifiable. All eyes were treated for cataract; that is, there were no refractive lens exchanges. In all cases, the Preop IOL power was determined using the Holladay post‐LASIK formula.

**Table 1:** Summary of Preop data

	Monofocal	Multifocal	*P*
*n* (Patients/eyes)	15/21	16/23	
Age†	65.9 ± 6.9(54–77)	58.8 ± 6.2(42–67)	0.01
Female/male	14/7	11/12	0.21
Years after LASIK†	12.5 ± 3.8(3–18)	12.1 ± 4.0(4–18)	0.71
Myopic/hyperopic LASIK	10/11	16/7	0.14
Preop refraction (D)† (spherical equivalent)	−0.50 ± 1.67(−4.87 to +3.25)	−0.68 ± 1.49(−4.00 to 1.25)	0.69
Preop average K (D)†	43.51 ± 3.11(39.83–52.50)	43.33 ± 2.7(37.78–48.15)	0.84
Preop IOL power (D)†	20.5 ± 1.9 (17.0 to 24.5)	20.1 ± 1.6 (17.0 to 22.5)	0.98

† Values are mean ± standard deviation (minimum to maximum).

D, diopter; IOL, intraocular lens; K, keratometry; LASIK, laser *in situ* keratomileusis; Preop, preoperative.

**Figure 1:**
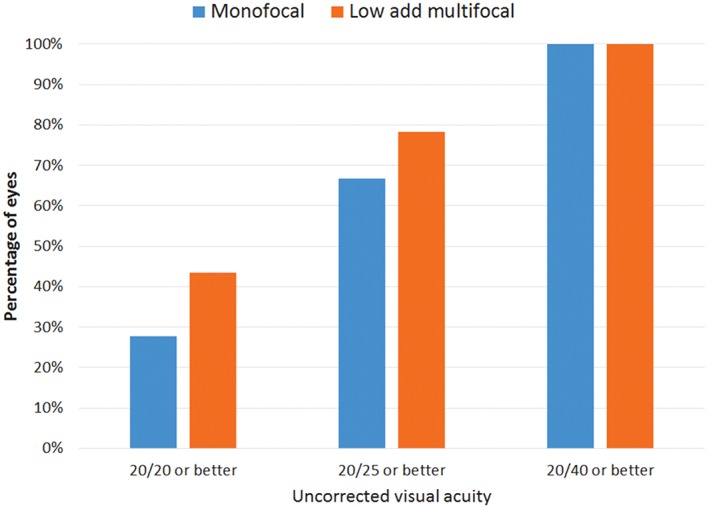
Postoperative uncorrected visual acuity.

Of perhaps most importance to patients is the uncorrected VA achieved. Figure 1 shows the cumulative uncorrected VA by lens type implanted. There was no statistically significant difference in the percentage of eyes with uncorrected distance VA of 20/25 or better between the IOL groups (chi‐square test, *P* = 0.41). The monofocal group includes only those eyes where the refractive target was plano (*n* = 18, as three eyes had a monovision target in the monofocal group).

The distant‐dominant multifocal IOL provided patients with slightly better intermediate than near vision. Of the eyes implanted with the multifocal IOL, 70% (16/23) had 20/20 or better best distance‐corrected VA at 60 cm, with no eye worse than 20/30. At 40 cm, just over half (52%, 12/23) of the eyes had a best distance‐corrected VA of 20/20 or better, again with no eye worse than 20/30.

**Figure 2:**
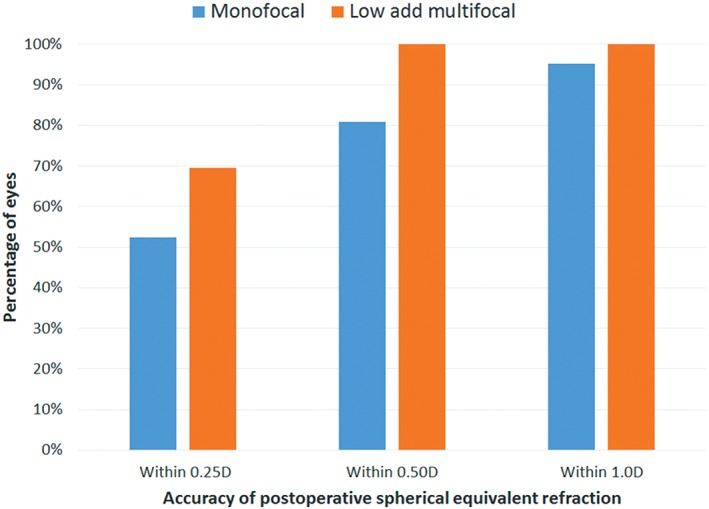
Accuracy of the postoperative spherical equivalent refraction (difference from target).

Figure 2 shows the cumulative accuracy of the spherical equivalent refraction, again by IOL group. The percentage of eyes with a refraction within 0.50D of intended was statistically significantly higher in the multifocal group (chi‐square test, *P* = 0.03). It was noted that the only four eyes with a spherical equivalent refractive error > 0.50D from target were eyes with previous hyperopic LASIK.

In 39% of cases (17/44), the Preop and IA power calculations suggested the same IOL power. In 11 of these cases, the surgeon (BF) implanted the suggested power, but in six others a lens of 0.5D higher was implanted, based on the IA prediction of a slight hyperopic residual refraction. In a further 19 cases, the IOL power suggested by IA was implanted. The IA influenced the IOL power chosen in five more cases, and in only three cases was the IOL power calculated preoperatively chosen when the IA power was different.

The prediction error is the difference between the postoperative refraction and the residual refraction expected from the IOL calculation (Preop or IA), the latter being adjusted for the power of the IOL that was implanted in the eye (if it was different from the calculated IOL power). A repeated measures ANOVA of the IA and Preop calculations showed a statistically significantly lower predicted residual error for the IA calculation (−0.11 ± 0.48 *vs*. −0.43 ± 0.48, *P* < 0.01). Note that the 0.32D difference is below the 0.50D step size in most IOL models. There was no significant effect of IOL type (*P* > 0.9).

Table 2 summarizes the individual results of the Preop and IA calculations, based on the implanted IOL and the postoperative refraction. The first column shows the difference in IOL power (if any) between the Preop and IA calculations. The columns are then categorized by which method appeared to be best. Looking at the cases where the Preop and IA calculations were not equal (columns 3 and 5), a chi‐square test showed that the IA results were not significantly more likely to be best (24/10 *vs*. 18/18, *P* = 0.08), but the low *P*‐value suggested a trend in that direction. There appeared to be a bias in the results, such that when IA suggested a higher IOL power, it was more likely to be best (17/4 *vs*. 11/11, *P* = 0.03).

**Table 2:** Categorization of actual results

Lens power difference	*n*	‘Better’ calculation (closer to postop)		
		IA	Equal†	Preop
IA > 1.00D higher	2	2		
IA 1.00D higher	5	3		2
IA 0.50D higher	15	12	1	2
Equal	17	7	7	3
IA 0.50D lower	4		2	2
IA 1.00D lower	1			1
All eyes	44	24	10	10

† Residual error difference between methods <0.10D.

IA, intraoperative aberrometry; Preop, preoperative; Postop, postoperative.

To determine whether the Preop formula used for IOL power calculation was a contributing factor in the differences mentioned above, IOL power calculations were repeated using the Barrett TrueK formula. A repeated measures ANOVA of the prediction error between IA and this formula showed no statistically significant difference (−0.11 ± 0.48 *vs*. −0.21 ± 0.61, *P* = 0.25). The type of IOL was not a statistically significant factor (*P* = 0.64).

Table 3 summarizes the individual results of the Preop and IA calculations in the theoretical instance where the Barrett TrueK formula was used to calculate Preop IOL power. The number of instances increased where the power calculated was the same (22 *vs*. 17 in Table 2), as did the number of instances where the calculation results were equal (15 *vs*. 10 in Table 2). As in Table 2, the data here showed no statistically significant difference in the number of best cases between Preop and IA (12/17 *vs*. 15/15, *P* = 0.44). When IA suggested a lower power, the Preop calculation was more often best, but this result was not statistically significant (10/2 *vs*. 6/6, *P* = 0.08). In contrast to the Holladay results mentioned above, there was no statistically significant difference in the number of best cases when the IOL power from IA was higher (5/3 *vs*. 4/4, *P* = 0.61).

**Table 3:** Categorization of results if Barrett TrueK formula was used preoperatively

Lens power difference	*n*	‘Better’ calculation (closer to postop)		
		IA	Equal†	Preop
IA > 1.00D higher	2	1		1
IA 1.00D higher	2	1		1
IA 0.50D higher	6	3	2	1
Equal	22	5	13	4
IA 0.50D lower	9	1		8
IA 1.00D lower	2			2
IA > 1.00D lower	1	1		
All eyes	44	12	15	17

† Residual error difference between methods <0.10D.

D, diopter; IA, intraoperative aberrometry, Preop, preoperative; Postop, postoperative.

Note that the Barrett TrueK formula appears to provide results that are less biased relative to the IA result, with 10 IA results higher and 12 IA results lower. This contrasts with Table 2, where the Holladay LASIK formula showed some bias, with 22 IA results higher and 5 IA results lower. The difference between the two Preop formulas was statistically significant (chi‐square test, *P* = 0.01).

## Discussion

It can be seen in this set of eyes that the clinical results achieved for distance VA and refraction with the distant‐dominant multifocal were equivalent to those achieved with the monofocal IOL. In addition, patients with the multifocal IOL had very good intermediate VA and good near VA. This is consistent with results described by Hayashi *et al*.^16^ who compared the same distant‐dominant multifocal and monofocal lens in a group of virgin eyes. In the current study, the percentage of eyes with a refraction within 0.50D of intended was statistically significantly higher in the multifocal group.

In the current study, 70% (16/23) of eyes implanted with the multifocal had 20/20 or better best distance‐corrected VA at 60 cm, with no eye worse than 20/30. This result is reasonably consistent with Alfonso *et al*.'s study who implanted the ReSTOR SN60D3 lens (Alcon Laboratories, Inc.) after hyperopic and myopic LASIK^12^, ^13^ and better than a 2010 United Kingdom Cataract National Dataset Electronic Multi‐centre Audit which reported that 95% of eyes, without visually significant ocular disease and likely with no history of LASIK, achieved 20/40 or better best‐corrected VA.^18^ In the current study, only four of 44 eyes or 9% had a spherical equivalent refractive error of >0.50D from target; these were eyes with previous hyperopic LASIK. With a similar population, Alfonso *et al*. noted that 27% of eyes were >0.50D away from the target refraction; these eyes also had previous hyperopic LASIK and were implanted with the ReSTOR SN60D3 lens (Alcon Laboratories, Inc.).^12^ Of note also is that Abulafia *et al*.^5^ who used the Barrett True‐K formula in patients with previous myopic LASIK or photorefractive keratectomy noted that 67% were within ±0.50D from target refraction while 91% of eyes in the current study were within ±0.50D from target. Results in the current study are also better than reported in other studies using other calculation methods where the highest reported percent of eyes within ±0.5D was 84%.^8^

IA influenced the surgeon's choice of lens in nearly all cases; this is reasonably consistent with the Ianchulev *et al*.'s study where the IA device influenced the surgeon's decision 68% of the time.^10^ The mean calculated residual error was lower for IA. However, the overall ratio of best results from IA relative to the Holladay LASIK formula was not statistically significantly different from random, except in the cases when IA predicted a higher lens power; in those instances, the number of best results was significantly better using the IA power. Superior results using the intraoperative aberrometer over other methods have been previously documented.^10^ The Holladay LASIK formula was much more likely to predict a lower power IOL, relative to IA. Using the Barrett TrueK formula for post‐LASIK eyes, there was no statistically significant difference between the IA and Preop calculation results.

A recognized limitation of the current study is the number of eyes analysed. Categorical statistical analyses are generally less sensitive than parametric analyses, all other things being equal. One complication here is that the basis for determining appropriate sample size depends on historical clinical data for the populations under study, and there are no extant articles related to the use of the ReSTOR 2.5 IOL (Alcon Laboratories, Inc.) in post‐LASIK eyes. The sample size calculation in the *Methods* section was our best estimate, although as noted, it was based on comparing parametric data. It can be safely stated that a larger data set would be beneficial, particularly for the categorical analysis of IA. Applying this analysis to larger data sets may provide definitive insights into the use of IA in post‐LASIK, and other, cases. The low numbers in the current study also meant that subset analyses were not possible, as the number in each subset was too low.

A second limitation was the retrospective nature of the study. One consequence of this was that while VA was evaluated, other measures of visual function such as contrast sensitivity were not. In addition, VA at different distances could not be compared between the monofocal and multifocal IOLs. This can be particularly interesting regarding intermediate vision. Near and intermediate VA testings are not routinely performed in patients receiving a monofocal IOL – a prospective study could include such measures and allow for comparisons.

In patients with a history of LASIK, a distant‐dominant multifocal lens is likely to provide improved intermediate and near VA while maintaining the same distance VA and refraction when compared with monofocal lenses. There was no apparent clinical benefit to the use of IA in the post‐LASIK eyes evaluated in this study, although a positive trend was evident. Larger prospective studies may be required to determine the patient‐specific value of IA in these cases.
